# CD3^bright^CD56^+^ T cells associate with pegylated interferon-alpha treatment nonresponse in chronic hepatitis B patients

**DOI:** 10.1038/srep25567

**Published:** 2016-05-13

**Authors:** Chuang Guo, Xiaokun Shen, Binqing Fu, Yanyan Liu, Yongyan Chen, Fang Ni, Ying Ye, Rui Sun, Jiabin Li, Zhigang Tian, Haiming Wei

**Affiliations:** 1 Institute of Immunology and The CAS Key Laboratory of Innate Immunity and Chronic Disease, School of Life Science and Medical Center, University of Science and Technology of China, Hefei 230027, People’s Republic of China; 2 Hefei National Laboratory for Physical Sciences at Microscale, University of Science and Technology of China, Hefei, Anhui, People’s Republic of China; 3 Department of Infectious Diseases, the First Affiliated Hospital of Anhui Medical University, Hefei, Anhui, People’s Republic of China

## Abstract

Chronic hepatitis B (CHB) infection is a serious and prevalent health concern worldwide, and the development of effective drugs and strategies to combat this disease is urgently needed. Currently, pegylated interferon-alpha (peg-IFNα) and nucleoside/nucleotide analogues (NA) are the most commonly prescribed treatments. However, sustained response rates in patients remain low, and the reasons are not well understood. Here, we observed that CHB patients preferentially harbored CD3^bright^CD56^+^ T cells, a newly identified CD56^+^ T cell population. Patients with this unique T cell population exhibited relatively poor responses to peg-IFNα treatment. CD3^bright^CD56^+^ T cells expressed remarkably high levels of the inhibitory molecule NKG2A as well as low levels of CD8. Even if patients were systematically treated with peg-IFNα, CD3^bright^CD56^+^ T cells remained in an inhibitory state throughout treatment and exhibited suppressed antiviral function. Furthermore, peg-IFNα treatment rapidly increased inhibitory TIM-3 expression on CD3^bright^CD56^+^ T cells, which negatively correlated with IFNγ production and might have led to their dysfunction. This study identified a novel CD3^bright^CD56^+^ T cell population preferentially shown in CHB patients, and indicated that the presence of CD3^bright^CD56^+^ T cells in CHB patients may be useful as a new indicator associated with poor therapeutic responses to peg-IFNα treatment.

The hepatitis B virus (HBV) infects more than 350 million people worldwide and is a major cause of chronic liver disease^1^. Both the innate and adaptive immune responses in the host regulate HBV infection^2^. In the innate immune response, hepatic natural killer (NK) cells exert their antiviral function against HBV infection by killing infected cells and producing high cytokine levels, which both promote the pathogenesis of viral hepatitis^3^. In the adaptive immune response, HBV-specific CD8^+^ T cells lyse infected hepatocytes and control viral infection; indeed, impaired CD8^+^ T cell activity is associated with the establishment of chronic HBV infection^4^. In addition, regulatory T cells are increased and have an immunosuppressive effect on HBV-specific T helper cells in chronic hepatitis B (CHB) patients^5^. The findings described above provide valuable information for understanding HBV pathogenesis and immune-evasion mechanisms. However, immune indexes that reflect the therapeutic efficacy of HBV treatments have not been so reliable, and other ways to evaluate therapeutic efficacy are needed.

Thus far, only three major clinical regimens to treat HBV are available: peg-IFNα, nucleoside/nucleotide analogues (NA), and the combination of peg-IFNα plus NA therapy^6^. Unlike HCV treatment that has yielded encouraging results, the effect of various therapies on HBV has been rather poor regardless of the treatment strategy. For instance, loss of hepatitis B e antigen (HBeAg)—a readout of reduced viral infectivity after treatment—occurs in only 30% of HBeAg-positive CHB patients treated with peg-IFNα, while the remaining 70% do not respond to treatment^7^. However, the underlying reason for this treatment resistance in HBV patients remains unknown.

A subset of the human T cell population expresses CD56, an NK cell surface marker. Generally, CD56^+^ T cells constitute approximately 10% of peripheral blood T cells and nearly 50% of liver T cells^8^,^9^. Upon stimulation, CD56^+^ T cells are activated, proliferate, and exhibit cytotoxicity in an MHC-unrestricted manner^10^,^11^. Notably, CD56^+^ T cells are a superior latent source of IFN-γ, which is considered to be a main mediator of antiviral responses^12^. As an abundant T cell subset in the liver, CD56^+^ T cells inhibit hepatic viral infection and replication, including HBV and HCV^13^,^14^. Moreover, CD56^+^ T cells are competent to treat a number of various infectious diseases^15^,^16^,^17^,^18^,^19^.

Despite this observed antiviral function, however, effector immune cells are always weaker in the context of HBV infection. We previously reported that TGFβ1 enrichment in HBV-persistent patients reduced NKG2D/2B4 expression on NK cells, leading to NK cell suppression^20^. In CHB patients, high NKG2A expression on NK cells decreased NK cell cytotoxicity^21^. Additionally, CHB patients reportedly harbor CD56^+^ T cells that display significantly increased inhibitory T cell immunoglobulin mucin-3 (Tim-3) expression over those from healthy controls, and this expression is further upregulated in patients with acute-on-chronic liver failure^22^. Tim-3 expression on CD56^+^ T cells also closely correlated with elevated serum ALT levels (a readout of liver injury) in CHB patients. Taken together, we speculate that CD56^+^ T cells may be in diminished antiviral status in CHB patients.

In order to understand the state of the immune system in CHB patients during HBV therapy, we evaluated new cases of untreated CHB patients who were systematically treated with peg-IFNα for 48 weeks. We identified that CHB patients could be classified into the following two different groups based on the intensity of CD3 expression on their CD56^+^ T cells: the CD3^bright^CD56^+^ T cell– and CD3^dim^CD56^+^ T cell–harboring CHB patient groups. Interestingly, a higher percentage of CHB patients (55/85, 64.7%) preferentially harbored the CD3^bright^CD56^+^ T cells than healthy controls (10/33, 30.3%). We further found that CD56^+^ T cells played an important role in the host response to peg-IFNα therapy and that the presence of peripheral CD3^bright^CD56^+^ T cells counted against host control of HBV and predicted poor therapeutic response. Indeed, CD3^bright^CD56^+^ T cells appeared to be both phenotypically and functionally inhibited. CD3^bright^CD56^+^ T cells rapidly upregulated Tim-3 expression during peg-IFNα treatment, which might explain the observed CD3^bright^CD56^+^ T cell dysfunction. Taken together, we provide a possible immunological explanation as to why a majority of CHB patients have a poor therapeutic response to peg-IFNα and present a new clinical outcome indicator that may serve as an auxiliary measurement of the efficacy of peg-IFNα treatment.

## Results

### A majority of CHB patients preferentially harbor a CD3^bright^CD56^+^ T cell population

**Figure 1:**
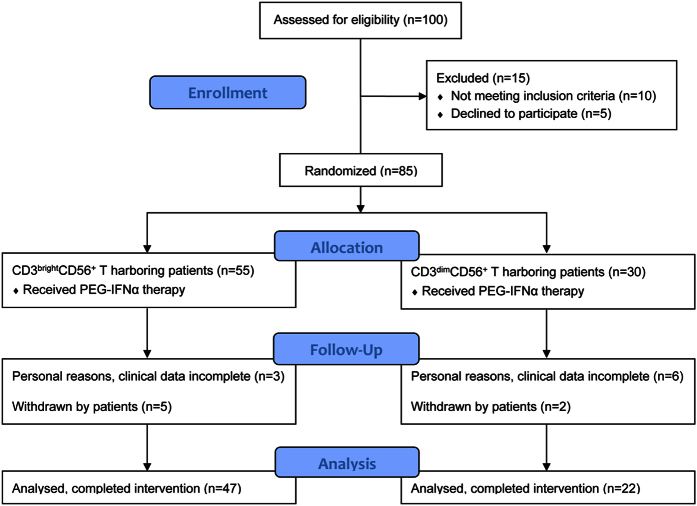
Patient CONSORT flowchart. Inclusion of patients from the initial study and reasons for not participating for those not enrolled in the clinical data.

**Figure 2:**
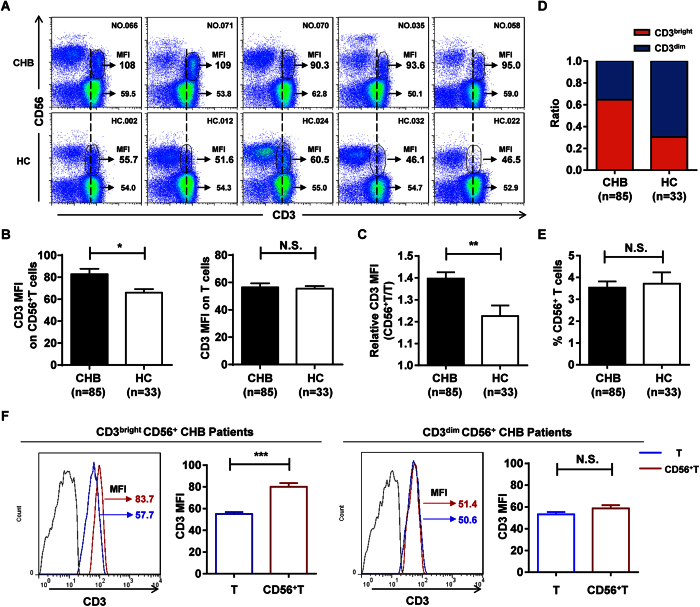
A majority of CHB patients preferentially harbor a CD3^bright^CD56^+^ T cell population. (**A**) Flow cytometric analysis of CD3 and CD56 double staining on CD3^+^CD56^+^ T cells in PBMCs from CHB patients (CHB, upper panel) and healthy controls (HC, lower panel). CD3 expression levels (MFI) on CD56^+^ T cells are indicated. (**B**) CD3 MFI on CD3^+^CD56^+^ T cells (left) and conventional CD3^+^CD56^−^ T cells (right) from CHB patients and healthy controls. (**C**) Relative CD3 MFI (CD56^+^/conventional CD56^−^ T) in CHB patients and healthy controls. (**D**) The frequency of individuals harboring CD3^bright^CD56^+^ (red) or CD3^dim^CD56^+^ (blue) T cells in CHB patients and healthy controls. (**E**) The percentage of CD56^+^ T cells in PBMCs from CHB patients and healthy controls. (**F**) CD3 MFI of T cells (blue) and CD56^+^ T cells (red) in CD3^bright^CD56^+^ (left) and CD3^dim^CD56^+^ (right) T cells in their respective CHB patient groups. Cumulative data for CD3 MFI were shown, respectively. Error bars, s.e.m. *P < 0.05, **P < 0.01, ***P < 0.001. N.S., not significant.

Based on previous studies, CD56^+^ T cells may represent a unique cell population with a significant immunoregulatory role in CHB patients. To further characterize these CD56^+^ T cells, we analyzed this cell population in live PBMC lymphocytes from 85 CHB patients and 33 healthy control individuals by flow cytometry (Fig. 1 and Supplementary Fig. S1A and S1B). We found that the mean fluorescence intensity (MFI) of CD3 expression was significantly higher on CD56^+^ T cells from CHB patients than from healthy controls, a difference not observed on conventional CD3^+^CD56^−^ T cells (Fig. 2A,B). We then assessed the ratio of CD3 MFI between CD56^+^ T and conventional CD3^+^CD56^−^ T cell populations (relative CD3 MFI) and found that CHB patients displayed significantly higher relative CD3 MFI than healthy controls (Fig. 2C), further demonstrating the higher CD3 MFI expression on CD56^+^ T cells in CHB patients.

As illustrated by the vertical dashed line in the representative patient samples shown in Fig. 2A, CHB patients clearly exhibited a higher overall CD3 MFI on their CD56^+^ T cells (called “CD3^bright^CD56^+^ T cells” here) than healthy controls, which harbored more of what we referred to as “CD3^dim^CD56^+^ T cells”. In order to distinguish them quantitatively, we analyzed the relative CD3 MFI from CHB patients based on that from healthy controls using the receiver operating characteristic (ROC) curve, and determined the cut-off (Supplementary Fig. S2). Using these designations, we classified each individual CHB patient and healthy control into one of two groups: one group that primarily harbored CD3^bright^CD56^+^ T cells and another group that primarily harbored CD3^dim^CD56^+^ T cells. Of the 85 total CHB patients enrolled in the study (Table 1), a majority (55/85, 64.7%) harbored the CD3^bright^CD56^+^ T cell population; in contrast, only 10/33 healthy controls (30.3%) harbored the CD3^bright^CD56^+^ T cell population. Indeed, the proportion of individuals harboring CD3^bright^CD56^+^ T cells was much higher in CHB patients than in healthy controls (Fig. 2D), even though the frequency of CD56^+^ T cells in PBMCs remained similar between CHB patients and healthy controls (Fig. 2E). We also confirmed that this difference in CD3 MFI expression occurred only in CD56^+^, but not conventional T cells in CHB patients (Fig. 2F). Taken together, these results suggest that CHB patients preferentially harbor CD3^bright^CD56^+^ T cells.

### CHB patients harboring CD3^bright^CD56^+^ T cells exhibit a poor therapeutic response to peg-IFNα

**Figure 3:**
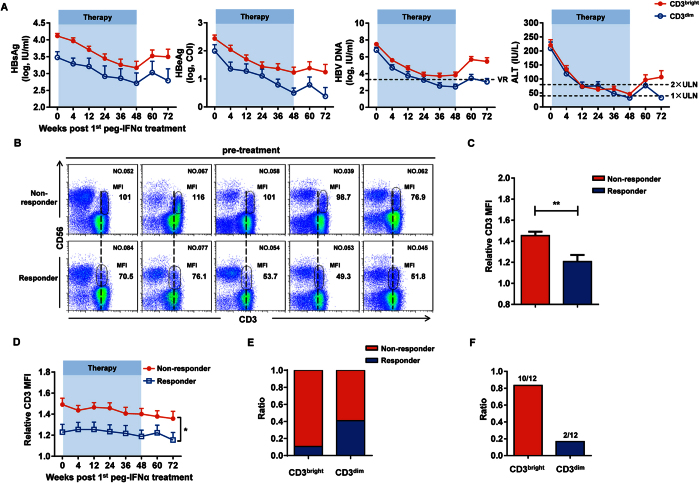
CHB patients harboring CD3^bright^CD56^+^ T cells exhibit a poor therapeutic response to peg-IFNα. (**A**) HBsAg, HBeAg, HBV DNA, and ALT levels in CHB patients harboring CD3^bright^CD56^+^ (red) or CD3^dim^CD56^+^ (blue) T cells at the indicated time points since peg-IFNα treatment. VR, virological response; ULN, upper limit of normal. (**B**) Immunofluorescent staining of CD56^+^ T cells in CHB patients before peg-IFNα treatment grouped by treatment effect. CD3 MFI of CD56^+^ T cells in nonresponders and responders is indicated. (**C**) Relative CD3 MFI values on CD56^+^ T cells from nonresponders (red) and responders (blue) CHB patients. (**D**) Relative CD3 MFI was evaluated in the different treatment effect groups at the indicated time points since peg-IFNα treatment. (**E**) The ratio of nonresponders (red) and responders (blue) harboring CD3^bright^CD56^+^ (red) or CD3^dim^CD56^+^ (blue) T cells. (**F**) The ratio of CD3^bright^CD56^+^ (red) to CD3^dim^CD56^+^ (blue) T cell populations in CHB patients who harbored HBsAg values >20000 IU/mL at 12 or 24 weeks of treatment. Error bars, s.e.m. *P < 0.05, **P < 0.01, ***P < 0.001.

We next determined whether there was a correlation between the presence of CD3^bright^CD56^+^ T cells in CHB patients and their response to therapy. Among the 69 from the total 85 CHB patients who had complete clinical data, we found that the clinical indexes of CHB patients harboring the CD3^bright^CD56^+^ T cell population were significantly higher at different time points after treatment (Fig. 3A). According to the EASL clinical practice guideline, response was defined as HBeAg loss with HBV DNA < 2000 IU/mL and ALT normalization at 24 weeks after treatment (72w), we compared the CD3 MFI on CD56^+^ T cells between the responders and nonresponders and found that the CD3 MFI on CD56^+^ T cells was significantly higher in nonresponders compared to responders (Fig. 3B); assessment of the relative CD3 MFI on CD56^+^ T cells between the responders and nonresponders revealed similar results (Fig. 3C). Furthermore, the relative CD3 MFI was notably higher in nonresponders since peg-IFNα treatment (Fig. 3D), and a larger proportion of CHB patients harboring CD3^bright^CD56^+^ T cells (42/47, 89.4%) were nonresponsive to the treatment than the patients harboring CD3^dim^CD56^+^ T cells (13/22, 59.1%) (Fig. 3E). As previously reported^23^, CHB patients exhibiting HBsAg > 20000 IU/mL at 12 or 24 weeks on peg-IFNα therapy were advised to discontinue therapy due to the low probability of achieving a sustained response. Within our patient population, 12/85 exhibited HBsAg > 20000 IU/mL at 12 or 24 weeks; 10 of these harbored the CD3^bright^CD56^+^ T cells (Fig. 3F) and maintained high HBsAg expression until the end of the therapy. Collectively, these results suggested that CHB patients harboring CD3^bright^CD56^+^ T cells exhibited a relatively poor therapeutic response to peg-IFNα treatment.

### CD3^bright^CD56^+^ T cells express low CD8 levels

**Figure 4:**
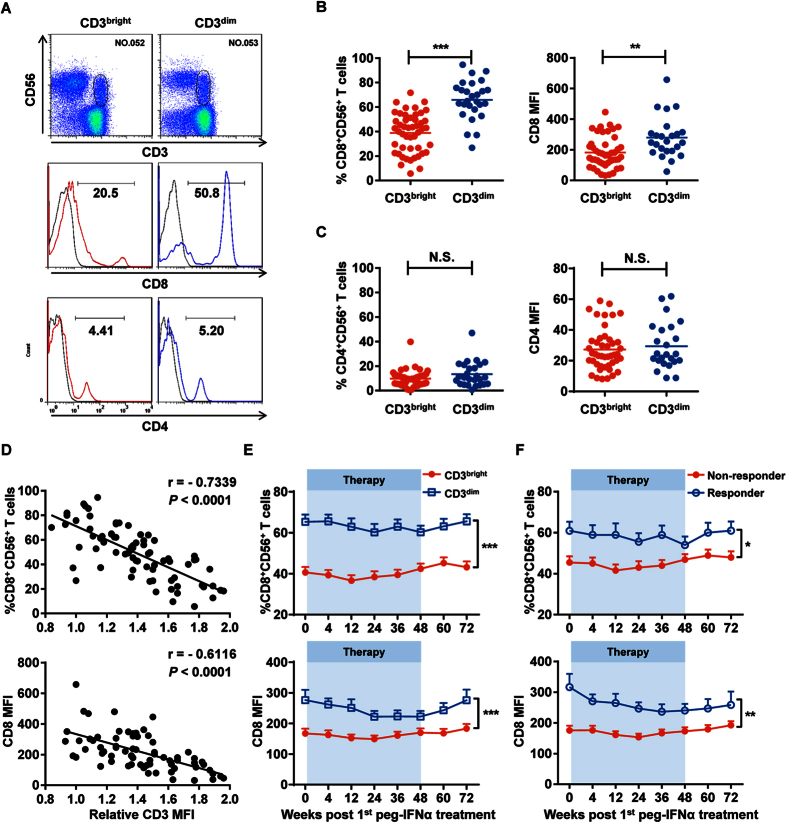
CD3^bright^CD56^+^ T cells expressed low CD8 levels. (**A**) CD8 (middle panel) and CD4 (bottom panel) expression on CD3^bright^CD56^+^ (red) and CD3^dim^CD56^+^ (blue) T cells from their respective CHB patient groups. Isotype controls (grey) were shown, respectively. Fluorescent antibodies against anti-CD3 and anti-CD56 were used to identify CD56^+^ T cells in CHB patient PBMCs (upper panel). (**B**) Cumulative data for frequency (left) and MFI (right) for the CD8 data shown in the middle panel of (**A**). (**C**) Cumulative data for frequency (left) and MFI (right) for the CD4 data shown in the lower panel of (**A**). (**D**) Inverse correlation between the frequency/MFI of CD8 expression and relative CD3 MFI on CD56^+^ T cells in CHB patients. Pearson’s correlation coefficient: r = −0.7339; *P* < 0.0001. (**E**) The frequency and MFI of CD8 expression on CD3^bright^CD56^+^ (red) and CD3^dim^CD56^+^ (blue) T cells in their respective CHB patient groups since peg-IFNα treatment. (**F**) The frequency and MFI of CD8 expression on CD56^+^ T cells from nonresponders (red) and responders (blue) since peg-IFNα treatment. Error bars, s.e.m. *P < 0.05, **P < 0.01, ***P < 0.001. N.S., not significant.

Based on the poor treatment effect we observed in CHB patients harboring CD3^bright^CD56^+^ T cells, we hypothesized that these CD3^bright^CD56^+^ T cells were dysfunctional compared to other CD56^+^ T cells. When comparing between the CHB patient groups harboring CD3^bright^CD56^+^ T cells or CD3^dim^CD56^+^ T cells, CD3^bright^CD56^+^ T cells remarkably exhibited lower CD8 expression than CD3^dim^CD56^+^ T cells (Fig. 4A,B); no differential CD4 expression was observed between the two groups (Fig. 4A,C). We also examined expression of the activation-related molecule CD69, maturation-related molecule CD57, and cytotoxicity-related molecule CD16, but did not observe any significant differences (Supplementary Fig. S3A–S3C).

We next compared CD8 and CD3 expression on CD56^+^ T cells and found that CD8 expression in CD56^+^ T cells negatively correlated with the relative CD3 expression in all CHB patients (Fig. 4D). Indeed, CD8 expression on CD56^+^ T cells in CHB patients harboring CD3^bright^CD56^+^ T cells was significantly lower than on those in CHB patients harboring CD3^dim^CD56^+^ T cells, even after peg-IFNα treatment (Fig. 4E). Meanwhile, no significant differences were observed on CD69, CD57, and CD16 expression during therapy (Supplementary Fig. S3D–S3F). With respect to the treatment effect, nonresponsders had the lowest frequency of CD8^+^CD56^+^ T cells, indicating that nonresponders might harbor more dysfunctional CD56^+^ T cells than responders (Fig. 4F). In summary, CD3^bright^CD56^+^ T cells expressed low CD8 levels throughout peg-IFNα treatment, and nonresponders harbored less CD8^+^CD56^+^ T cells than responders.

### CD3^bright^CD56^+^ T cells express high inhibitory NKG2A and CD94 receptors

**Figure 5:**
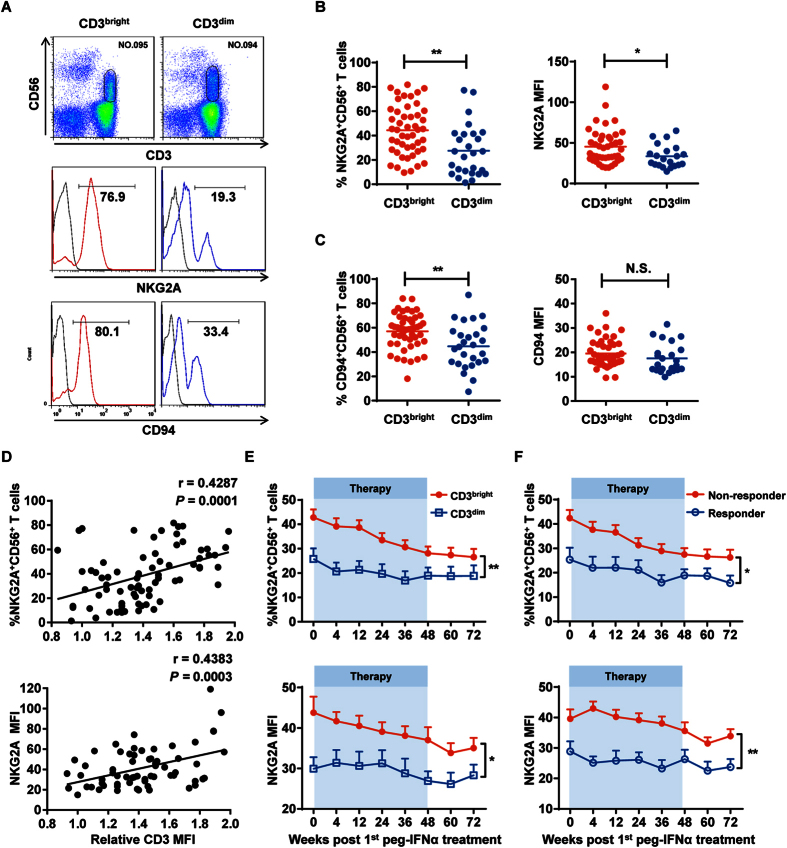
CD3^bright^CD56^+^ T cells expressed high inhibitory NKG2A receptor. (**A**) NKG2A (middle panel) and CD94 (bottom panel) expression on CD3^bright^CD56^+^ (red) or CD3^dim^CD56^+^ (blue) T cells from their respective CHB patient groups. Isotype controls (grey) were shown, respectively. Fluorescent antibodies against anti-CD3 and anti-CD56 were used to identify CD56^+^ T cells in CHB patient PBMCs (upper panel). (**B**) Cumulative data for frequency (left) and MFI (right) for the NKG2A data shown in the middle panel of (**A**). (**C**) Cumulative data for frequency (left) and MFI (right) for the CD94 data shown in the lower panel of (**A**). (**D**) Positive correlation between the frequency / MFI of NKG2A expression and relative CD3 MFI on CD56^+^ T cells in CHB patients. Pearson’s correlation coefficient: r = 0.4287; *P* = 0.0001. (**E**) The frequency and MFI of NKG2A expression on CD3^bright^CD56^+^ (red) and CD3^dim^CD56^+^ (blue) T cells in their respective CHB patient groups since peg-IFNα treatment. (**F**) The frequency and MFI of NKG2A expression on CD56^+^ T cells from nonresponders (red) and responders (blue) since peg-IFNα treatment. Error bars, s.e.m. *P < 0.05, **P < 0.01. N.S., not significant.

To next explore whether CD3^bright^CD56^+^ T cells were undergoing any inhibition, we assessed their phenotypic expression of inhibitory molecules. Remarkably, within the corresponding CHB patient groups, CD3^bright^CD56^+^ T cells displayed higher expression of the inhibitory molecule NKG2A compared to CD3^dim^CD56^+^ T cells (Fig. 5A,B); similar results were observed for its heterodimer CD94 (Fig. 5A,C). In contrast, no significant differences in expression of other inhibitory molecules, such as PD-1 and CTLA-4, were detected (Supplementary Fig. S4A and S4B). Furthermore, NKG2A expression on CD56^+^ T cells exhibited a remarkable positive correlation with CD3 expression in all CHB patients (Fig. 5D). During peg-IFNα therapy, NKG2A, but not PD-1 or CTLA-4, maintained a higher expression on CD3^bright^CD56^+^ T cells compared to CD3^dim^CD56^+^ T cells (Fig. 5E, Supplementary Fig. S4C and S4D). With respect to the treatment effect, nonresponders had the highest frequency of NKG2A^+^CD56^+^ T cells compared to responders (Fig. 5F). Interestingly, NKG2A and CD94 expression on CD56^+^ T cells exhibited a remarkable negative correlation with CD8 expression in CHB patients harboring CD3^bright^CD56^+^ T cells, but not in patients harboring CD3^dim^CD56^+^ T cells or healthy controls (Supplementary Fig. S5A–S5C). Taken together, our results showed that CD3^bright^CD56^+^ T cells expressed high levels of the inhibitory heterodimers NKG2A and CD94 throughout peg-IFNα treatment, and nonresponders harbored more NKG2A^+^CD56^+^ and CD94^+^CD56^+^ T cells than responders.

### CD3^bright^CD56^+^ T cells exhibit dysfunctional antiviral responses

**Figure 6:**
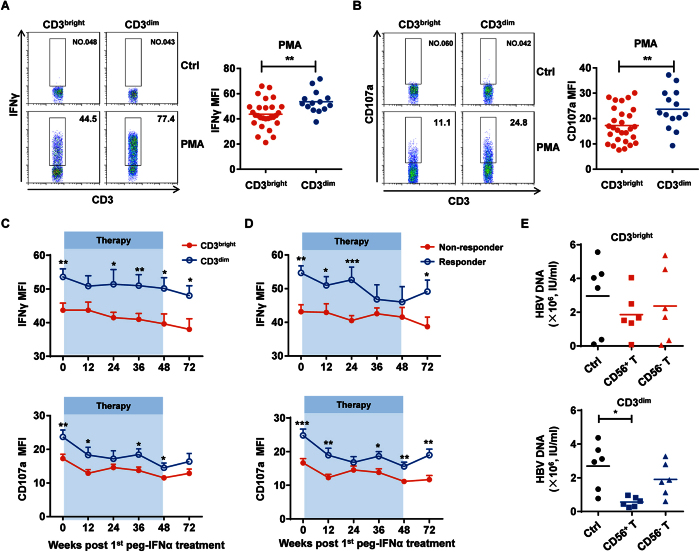
CD3^bright^CD56^+^ T cells exhibit dysfunctional antiviral responses. (**A**) Freshly isolated PBMCs from CHB patients before treatment were stimulated with or without PMA and ionomycin for 4 h, and intracellular staining of IFNγ was determined using flow cytometry by gating on CD3^+^CD56^+^ cells. Statistical data are shown for the compiled data. (**B**) CD107a expression was detected since PMA and ionomycin stimulation. Statistical data are shown. (**C**) IFNγ (upper) and CD107a (lower) expression in CD56^+^ T from CHB patients harboring either the CD3^bright^CD56^+^ (red) or CD3^dim^CD56^+^ (blue) T cells at the indicated time points since peg-IFNα treatment. (**D**) IFNγ (upper) and CD107a (lower) expression on CD56^+^ T cells from nonresponders (red) and responders (blue) since peg-IFNα treatment. (**E**) Co-culture of HBV-carrying-HepG2.2.15 cells with purified CD3^bright^CD56^+^ (upper) or CD3^dim^CD56^+^ (lower) T cells at a 1:10 ratio via transwell system. HBV DNA were determined. Error bars, s.e.m. *P < 0.05, **P < 0.01, ***P  <  0.001.

IFNγ reportedly plays an important role in the host antiviral response, and peg-IFNα therapy is thought to potentiate IFNγ secretion by antiviral effector cells^24^,^25^. Based on the inhibitory phenotype we observed on CD3^bright^CD56^+^ T cells, we hypothesized that these T cells had reduced antiviral function and tested this idea by evaluating their ability to secrete IFNγ. Intracellular staining of IFNγ on CD56^+^ T cells from CHB patients in our cohort by flow cytometry revealed that CD3^bright^CD56^+^ T cells produced lower IFNγ levels compared to CD3^dim^CD56^+^ T cells (Fig. 6A and Supplementary Fig. S6A). Moreover, CD3^bright^CD56^+^ T cell degranulation was also lower than CD3^dim^CD56^+^ T cells, as CD107a expression by CD3^bright^CD56^+^ T cells was less than that compared to CD3^dim^CD56^+^ T cells (Fig. 6B and Supplementary Fig. S6B). During peg-IFNα therapy, IFNγ and CD107a expression in CD3^bright^CD56^+^ T cells were consistently lower throughout the treatment period (Fig. 6C). With respect to the treatment effect, nonresponders had the lower frequency of IFNγ^+^CD56^+^ and CD107a^+^CD56^+^ T cells compared to responders (Fig. 6D). To examine the effect of CD56^+^ T cells suppressing HBV replication in human hepatocytes *in vitro*, HBV-producing hepatoma cell line HepG2.2.15 was co-cultured with CD3^bright^CD56^+^ or CD3^dim^CD56^+^ T cells. The level of HBV DNA was reduced to a larger extent in HepG2.2.15 which was co-cultured with CD3^dim^CD56^+^ T cells, suggesting that the anti-HBV efficiency of CD3^bright^CD56^+^ T cells was much lower compared to CD3^dim^CD56^+^ T cells. (Fig. 6E). Thus, our results suggested that CD3^bright^CD56^+^ T cells had dysfunctional antiviral activity.

### Tim-3 upregulation correlates with CD3^bright^CD56^+^ T cell dysfunction during peg-IFNα treatment

**Figure 7:**
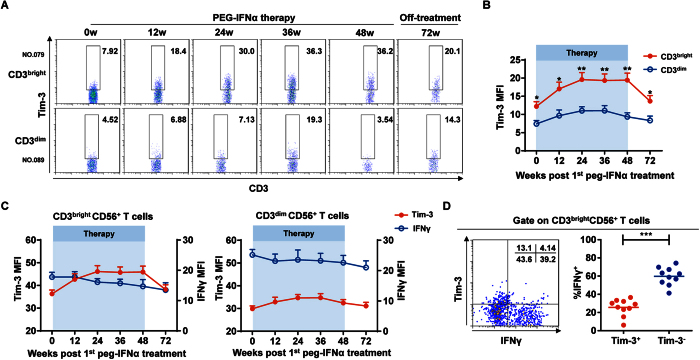
TIM-3 upregulation correlates with CD3^bright^CD56^+^ T cell dysfunction during peg-IFNα treatment. (**A**) TIM-3 expression on CD56^+^ T cells from CHB patients harboring either the CD3^bright^CD56^+^ (upper) or CD3^dim^CD56^+^ (lower) T cells at the indicated time points since peg-IFNα treatment. (**B**) Cumulative data for TIM-3 expression on CD56^+^ T cells shown in (**A**). (**C**) TIM-3 expression (red) and IFNγ production (blue) in CD3^bright^CD56^+^ (left) and CD3^dim^CD56^+^ (right) T cells in their respective CHB patient groups since peg-IFNα therapy. (**D**) IFNγ production in Tim3^+^CD56^+^ or Tim3^−^CD56^+^ T cells. Error bars, s.e.m. *P < 0.05, **P < 0.01, ***P < 0.001.

Tim-3 reportedly suppresses several effector cells in CHB patients^26^,^27^. We therefore speculated that Tim-3 might also participate in inhibiting CD3^bright^CD56^+^ T cell function. Although Tim-3 expression was similar between CD3^bright^CD56^+^ and CD3^bright^CD56^+^ T cells before peg-IFNα treatment, Tim-3 expression rapidly increased on CD3^bright^CD56^+^, but not CD3^dim^CD56^+^, T cells during peg-IFNα treatment (Fig. 7A,B). Moreover, Tim-3 expression on CD3^bright^CD56^+^ T cells negatively correlated with IFNγ secretion, whereas Tim-3 and IFNγ expression exhibited a similar pattern on CD3^dim^CD56^+^ T cells (Fig. 7C). Indeed, we confirmed the lower frequency of IFNγ on Tim3^+^CD56^+^ T cells compared to that on Tim3^-^CD56^+^ T cells (Fig. 7D). Taken together, the negative correlation between Tim-3 upregulation and IFNγ secretion on CD56^+^ T cells might provide a plausible explanation for the observed CD3^bright^CD56^+^ T cell dysfunction during peg-IFNα treatment in CHB patients harboring this CD56^+^ T cell population (Supplementary Fig. S7).

## Discussion

Previous reports found that CD56^+^ T cells played a key role in determining the outcome of acute HCV infection^28^. However, the sustained role of CD56^+^ T cells in regulating HBV infection during peg-IFNα treatment is largely unknown. Our results revealed that a majority of CHB patients preferentially harbor CD3^bright^CD56^+^ T cells compared to healthy controls and that the presence of this population could act as an auxiliary predictor of peg-IFNα efficacy during the treatment period. Based on the intensity of CD3 MFI on CD56^+^ T cells, we are the first to reveal two phenotypically and functionally different CD56^+^ T cell populations and that the CD3^bright^CD56^+^ T cell population occurs more frequently in CHB patients than CD3^dim^CD56^+^ T cells. CD3^bright^CD56^+^ T cells were phenotypically and functionally in an inhibited state, which included higher inhibitory NKG2A/CD94 expression as well as lower CD8 expression. Moreover, CD3^bright^CD56^+^ T cells exhibited lower IFNγ secretion, considered to be the main driving force of the antiviral response against HBV. Peg-IFNα treatment did not improve the CD3^bright^CD56^+^ T cell inhibitory state, as high NKG2A/CD94 and low CD8 expression was maintained during treatment. Consistent with the inhibitory phenotype and function of CD3^bright^CD56^+^ T cells, CHB patients harboring these CD3^bright^CD56^+^ T cells had relatively poor responses to peg-IFNα treatment. Finally, our data demonstrating that Tim-3 expression was rapidly upregulated on CD3^bright^CD56^+^ T cells during peg-IFNα treatment might provide a mechanistic understanding of the observed T cell dysfunction in CHB patients.

Previous clinical studies suggested the use of several viral outcome predictors to show an adjunctive measure of HBeAg seroconversion for evaluating peg-IFNα treatment efficacy in HBV patients^23^,^29^,^30^. However, these predictors were obtained several weeks after treatment began, whereas the ideal predictor would be assessed before treatment to identify patients that were not likely to respond to therapy. Here, our results suggest that the presence of the CD3^bright^CD56^+^ T cell population in CHB patients can be used as a novel immunological predictor that can be easily detected before clinical peg-IFNα treatment, and it can also provide a new auxiliary indicator for evaluating the efficacy of peg-IFNα treatment of CHB patients during therapy.

A certain proportion of CHB patients in this study were assigned to receive peg-IFNα-2b in combination with adefovir dipivoxil (ADV). To investigate whether the treatment regimen affects the frequency of CD3^bright^ CD56^+^ T cells, we compared the relative CD3 MFI of CD56^+^ T cells from patients treated with either PEG-IFNα or PEG-IFNα plus ADV at the indicated time points since peg-IFNα treatment. However, no significant differences were detected at any time point since peg-IFNα treatment (Supplementary Fig. S8). In addition, CD3^bright^CD56^+^ T cells expressed lower CD8 levels and higher NKG2A levels than CD3^dim^CD56^+^ T cells regardless of treatment regimens (Supplementary Fig. S9A and S9B). Thus, treatment regimen does not affect the frequency of CD3^bright^ CD56^+^ T cells.

Tim-3 functions as a negative regulatory receptor for both innate and adaptive immune effector cells. It is already known to mediate NK cell suppression in HBV infection^31^. In terms of regulating T cells in HBV infection, Tim-3 upregulation in CHB patients contributes to T cell inhibition and deletion, and Tim-3 blockade enhances the antiviral function of HBV-specific T cells^32^. Our present results showed that Tim-3 expression rapidly increased on CD3^bright^CD56^+^ T cells in CHB patients after peg-IFNα treatment and closely correlated with the decrease in antiviral IFNγ secretion. Thus, based on the evidence here and in previous studies, we speculate that the upregulation of Tim-3 on CD3^bright^CD56^+^ T cells upon peg-IFNα treatment results in the poor therapeutic effect of peg-IFNα on CHB patients harboring CD3^bright^CD56^+^ T cells.

In conclusion, our findings identify a previously unrecognized role of CD3^bright^CD56^+^ T cells in CHB patients during peg-IFNα treatment. The inhibitory state of CD3^bright^CD56^+^ T cells and the rapidly increased Tim-3 expression during therapy can provide an explanation for the poor therapeutic effect of peg-IFNα treatment in a majority of CHB patients. Moreover, these findings present a new auxiliary clinical indicator for evaluating the efficacy of peg-IFNα therapy in CHB patients.

## Methods

### Patients, healthy controls, and peg-IFNα treatment

Data presented in this study were obtained from 85 new cases of HBeAg-positive CHB patients (Table 1) and 33 healthy controls. All HBV samples were collected at the Department of Infectious Diseases from June 2012 to July 2014, and peripheral blood samples from healthy controls were collected at the Department of Clinical Laboratory (the First Affiliated Hospital of Anhui Medical University).

The peripheral blood samples of all HBV patients in the study had detectable baseline serum HBV DNA (>2 × 10^4^ IU/ml) and elevated serum ALT (>2 × ULN and <10 × ULN). Reasons for exclusion were as follows: presence of serum antibodies against HCV, HDV or HIV, liver cirrhosis, acquired or inherited liver disease, prior antiviral or immunomodulatory treatment.

CHB patients were randomly assigned to two different treatment groups: 1.5 μg/kg PEG-IFNα-2b (PegIntron, Schering-Plough, Kenilworth, NJ, USA) weekly or peg–IFNα-2b weekly in combination with 10 mg adefovir dipivoxil (ADV, Hepsera, Gilead Sciences, Foster City, CA, USA) daily. Therapeutic regimens continued for 48 weeks and were followed by a 24-week post-treatment observation period.

### Ethics Statement

Informed consent obtained from all patients donating blood samples was written of their own accord, and the study was approved by the ethics committee of the First Affiliated Hospital of Anhui Medical University (Grant No. K2010003) and was carried out in accordance with the approved guidelines. This clinical research was also enrolled in the Chinese Clinical Trial Registry (Clinical trial registration number: ChiCTR-TRC-12002226; the URL: http://www.chictr.org.cn/en/; Registered on April 17, 2012).

### Reagents and antibodies

The following antibodies were purchased from BD Pharmingen: APC-Cy7–anti-CD3, Alexa-647–anti-CD56, PE-Cy7–anti-CD8, PerCP-Cy5.5–anti-CD4, FITC–anti-CD94, FITV–anti-IFN-γ, and PE–anti-CD107a. The following antibodies were purchased from R&D Systems: PE–anti-NKG2A and PE–anti-Tim-3. Isotype-matched negative control antibodies were all purchased from BD Pharmingen. Red blood cell (RBC) lysis buffer was purchased from BioLegend.

### Immunostaining

After removing plasma from blood samples, the indicated fluorochrome-conjugated monoclonal antibodies were added to 100 μL whole blood. The red blood cells were lysed with RBC lysis buffer using standard procedures. Stained cells were collected using an LSR II flow cytometer (BD Biosciences), and data were analyzed using FlowJo analysis software 7.6.1 (Tree Star, Inc.). Human CD56^+^ T cells were gated as CD3^+^ CD56^+^ lymphocytes for further analysis.

### Intracellular IFN-γ and CD107a detection

PBMCs (5 × 10^6^/mL) from CHB patients were stimulated with PMA (30 ng/mL, Sigma) and ionomycin (1 μg/mL, Calbiochem). Monensin (2.5 μg/mL, Sigma) was added to prevent secretion of the induced cytokines into the supernatant, and cells were cultured at 37 °C and 5% CO_2_ for 4 h. Cells were then harvested and labeled with Alexa-647–anti-CD56 and APC-Cy7–anti-CD3 for 30 min at 4 °C. After fixation and permeabilization, cells were stained with FITC-anti–IFN-γ or isotype negative control for 1 h at 4 °C. Degranulating cells were detected by culturing cells in the presence of PE–anti-CD107a for 4 h before surface staining. Cells were collected and analyzed after being washed with permeabilization buffer.

### Primary CD56^+^ T cell purification

CD56^+^ T cells were enriched from peripheral blood mononuclear cells of CHB patients with CD3^+^ CD56^+^ T Cell Isolation Kit (Miltenyi Biotec). The purity of CD56^+^ T cells measured by flow cytometry was greater than 95%. Purified CD56^+^ T or CD56^-^ T cells were cultured in RPMI 1640 containing 10% fetal bovine serum (HyClone).

### CD56^+^ T cells stimulation *in vitro*

1 × 10^6^ fresh PBMCs from CHB patients were stimulated with or without recombinant human IL-12 (rhIL-12; 10 ng/mL, Peprotech) for 16 h, and IFNγ production by CD56^+^ T cells was determined as above. For CD107a detection after stimulation *in vitro*, 1 × 10^6^ human PBMCs and 1 × 10^5^ cells of the erythroleukemia cell line K562 (American Type Culture Collection) were co-cultured for 4 h. Degranulation was detected with a PE-labelled anti-CD107a.

### Co-Culture of HepG2.2.15 cell line with CD56^+^ T cells

CD56^+^ T cells were activated by a standard T Cell Activation/Expansion Kit (Miltenyi Biotec) according to the manufacturer’s instructions. Hepatoma cell line HepG2.2.15 was co-cultured with CD56^+^ T cells via a transwell system at a 1:10 ratio. CD56^−^ T cells were used as a control in the experiments.

### Virological assessment

Serum HBV DNA levels were quantified using Roche COBAS TaqMan HBV Test (F. Hoffmann-La Roche Ltd., Basel Switzerland), with the lower detection limit threshold set at 20 IU/ml. Serum HBsAg levels were quantified using Roche Elecsys HBsAg II quant assay (Roche Diagnostics, China; the lower detection limit threshold was 0.05 IU/mL). The quantification of serum HBeAg and anti-HBe levels were performed based on the cut-off index (COI) by Roche Elecsys 2010 immunoanalyzer (Roche Diagnostics, China). ALT was measured locally in accordance with standard procedures. HBV genotype was performed using genotype-specific primers^33^.

Response to treatment was defined as a composite endpoint of HBeAg loss with HBV DNA level <2,000 IU/mL (~10,000 copies/mL) and ALT normalization at the end of 72 weeks (48 weeks of treatment and 24 weeks of follow-up)^30^.

### Statistics

A two-tailed Student’s *t*-test was used to statistically compare differences between two groups. A two-way ANOVA test was used to statistically compare differences among multiple groups at different time points after treatment. Data are expressed as mean ± standard error of the mean (s.e.m.). Statistical analyses were performed with Prism 6 (GraphPad Software, Inc.) or SPSS Statistics version 22 (IBM). Differences achieving values of *P* < 0.05 were considered to be statistically significant.

## Additional Information

**How to cite this article**: Guo, C. *et al*. CD3^bright^ CD56^+^ T cells associate with pegylated interferon-alpha treatment nonresponse in chronic hepatitis B patients. *Sci. Rep*. **6**, 25567; doi: 10.1038/srep25567 (2016).

## Supplementary Material

Supplementary Information

## Figures and Tables

**Table 1:** Characteristics of the Study Cohort.

CHB patients Characteristics	CD3^bright^CD56^+^ T harboring	CD3^dim^CD56^+^ T harboring
Total number	55	30
Mean age (SD)	28 (7)	28 (6)
Male	36 (65%)	21 (70%)
Peg-IFNα	26 (47%)	17 (57%)
Peg-IFNα plus ADV	29 (53%)	13 (43%)
**Pre-treatment**		
HBsAg (SD), log IU/mL	4.21 (0.52)	3.59 (0.81)
HBeAg (SD), log COI	2.63 (0.64)	2.30 (0.91)
HBV DNA (SD), log IU/mL	7.42 (1.22)	6.82 (1.53)
ALT (SD), U/L	215 (130)	227 (116)
HBV genotype	B (27), C (28)	B (15), C (15)
