# Supplementary material for: CD3brightCD56+ T cells associate with pegylated interferon-alpha treatment nonresponse in chronic hepatitis B patients
Source: Sci Rep. 2016 May 13;6:25567. doi: 10.1038/srep25567 (PMC4865958; doi:10.1038/srep25567)
Supplement: Supplementary Information [file srep25567-s1.doc]

**Supplementary Information**

**CD3^bright^CD56^+^ T cells associate with pegylated interferon-alpha** **treatment nonresponse in chronic hepatitis B patients**

Chuang Guo^1^, Xiaokun Shen^1^, Binqing Fu^1, 2^, Yanyan Liu^3^, Yongyan Chen^1^, Fang Ni^1^, Ying Ye^3^, Rui Sun^1, 2^, Jiabin Li^3*^, Zhigang Tian^1, 2*^, Haiming Wei^1, 2*^

**Affiliations**:

^1^ Institute of Immunology and The CAS Key Laboratory of Innate Immunity and Chronic Disease, School of Life Science and Medical Center, University of Science and Technology of China, Hefei 230027.

^2^ Hefei National Laboratory for Physical Sciences at Microscale, University of Science and Technology of China, Hefei, Anhui, People’s Republic of China.

^3^ Department of Infectious Diseases, the First Affiliated Hospital of Anhui Medical University, Hefei, Anhui, People’s Republic of China.

**Supplementary Methods**

**CD56^+^ T cells stimulation in vitro**

1**×**10^6^ Fresh PBMCs from CHB patients were stimulated with or without recombinant human IL-12 (rhIL-12; 10 ng/mL, Peprotech) for 16 h, and IFNγ production by CD56^+^ T cells was determined as above. For CD107a detection after stimulation in vitro, 1**×**10^6^ human PBMCs and 1**×**10^5^ cells of the erythroleukemia cell line K562 (American Type Culture Collection) were co-cultured for 4 h, degranulation was detected with a PE-labelled anti-CD107a-Ab.

**Supplementary Figures**

**
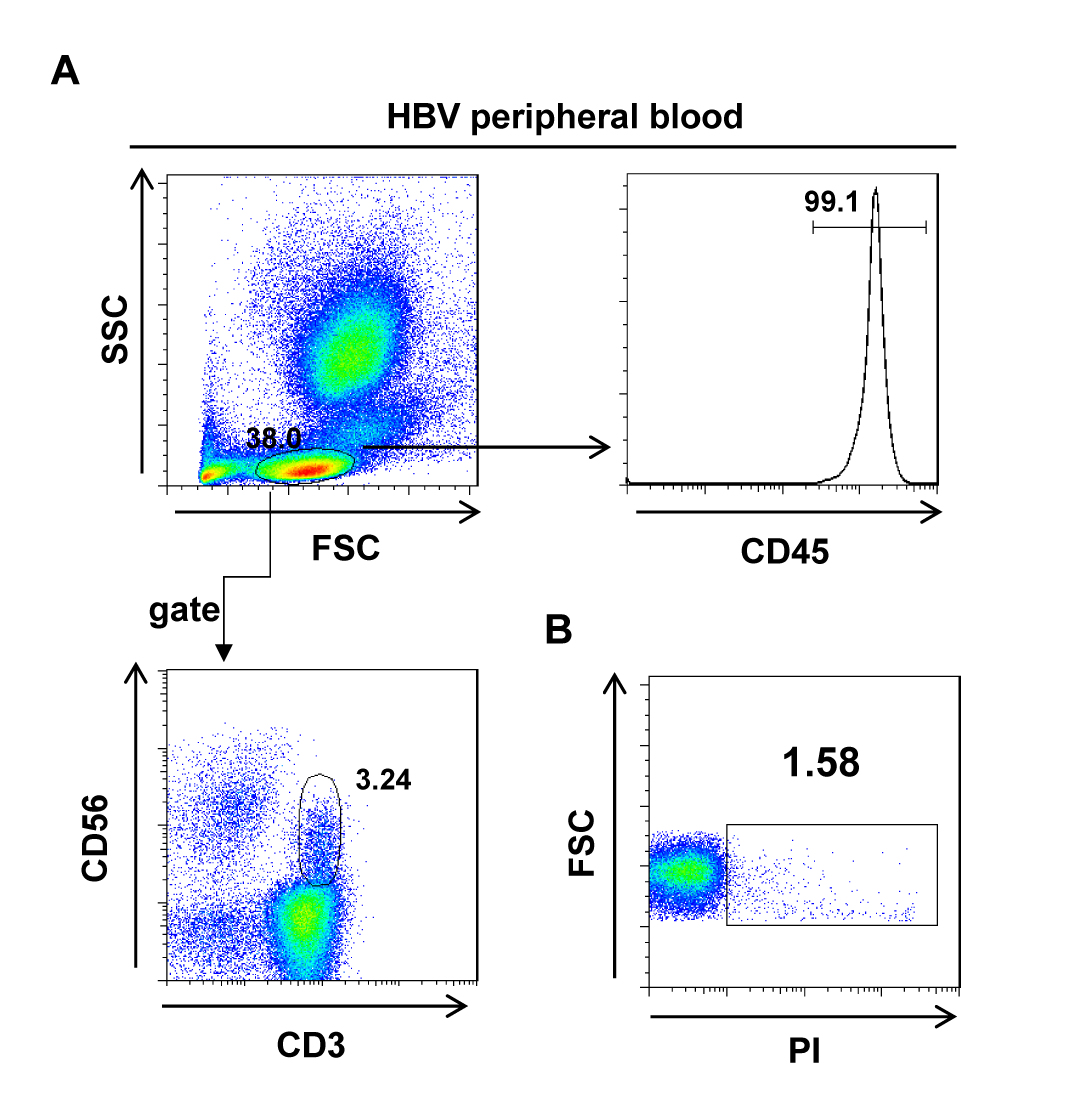
**

**Supplementary Fig. S1. Gating Strategy.**


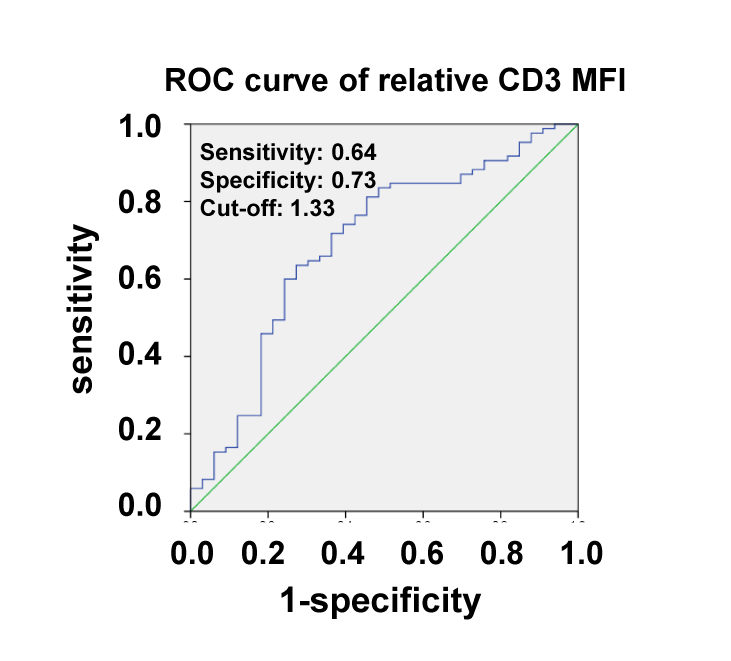


**Supplementary Fig. S2. ROC curve of relative CD3 MFI.**

**
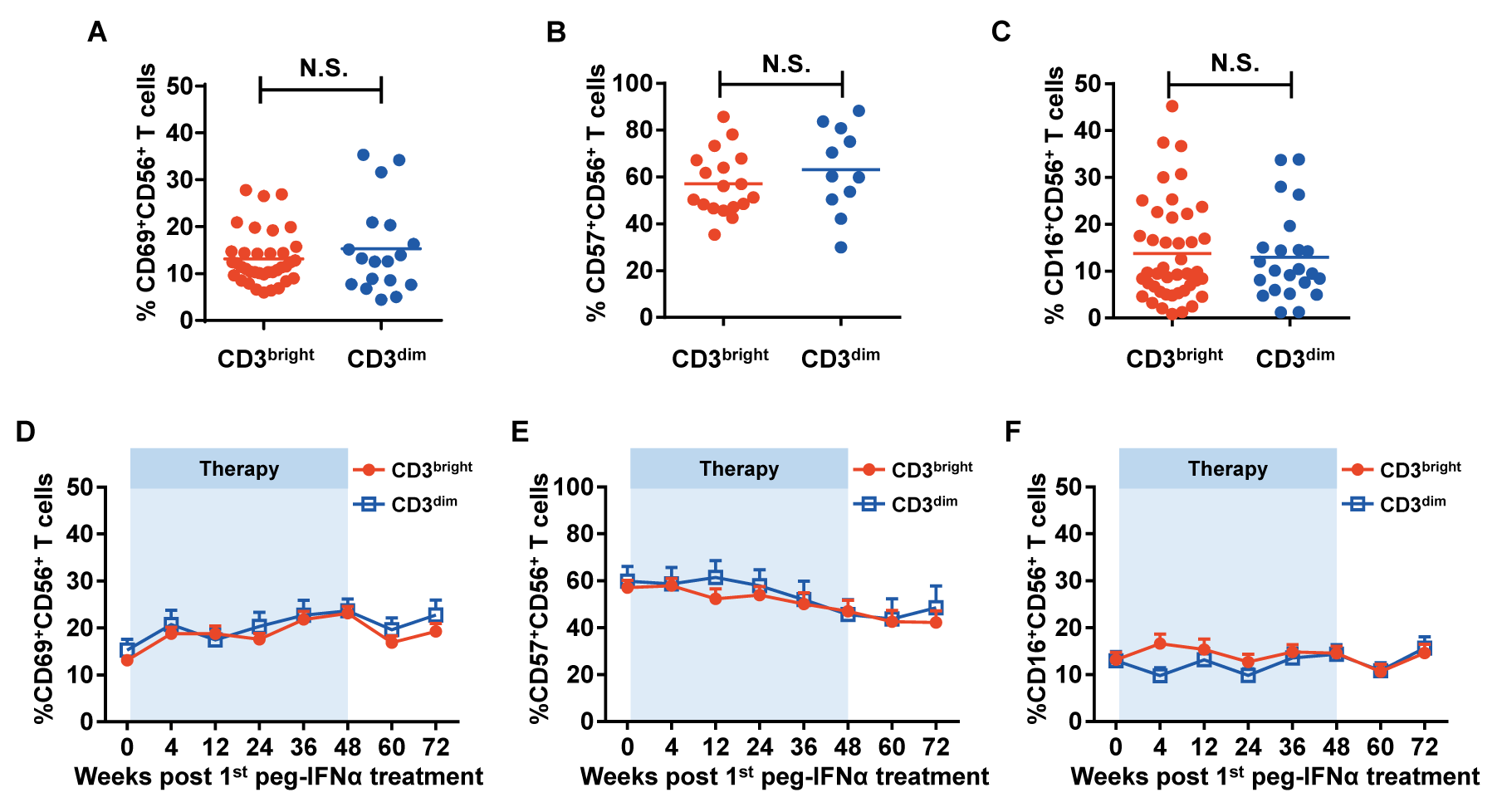
**

**Supplementary Fig. S3. CD3^bright^CD56^+^ T cells expressed similar levels of other functional molecules compared to CD3^dim^CD56^+^ T cells.**


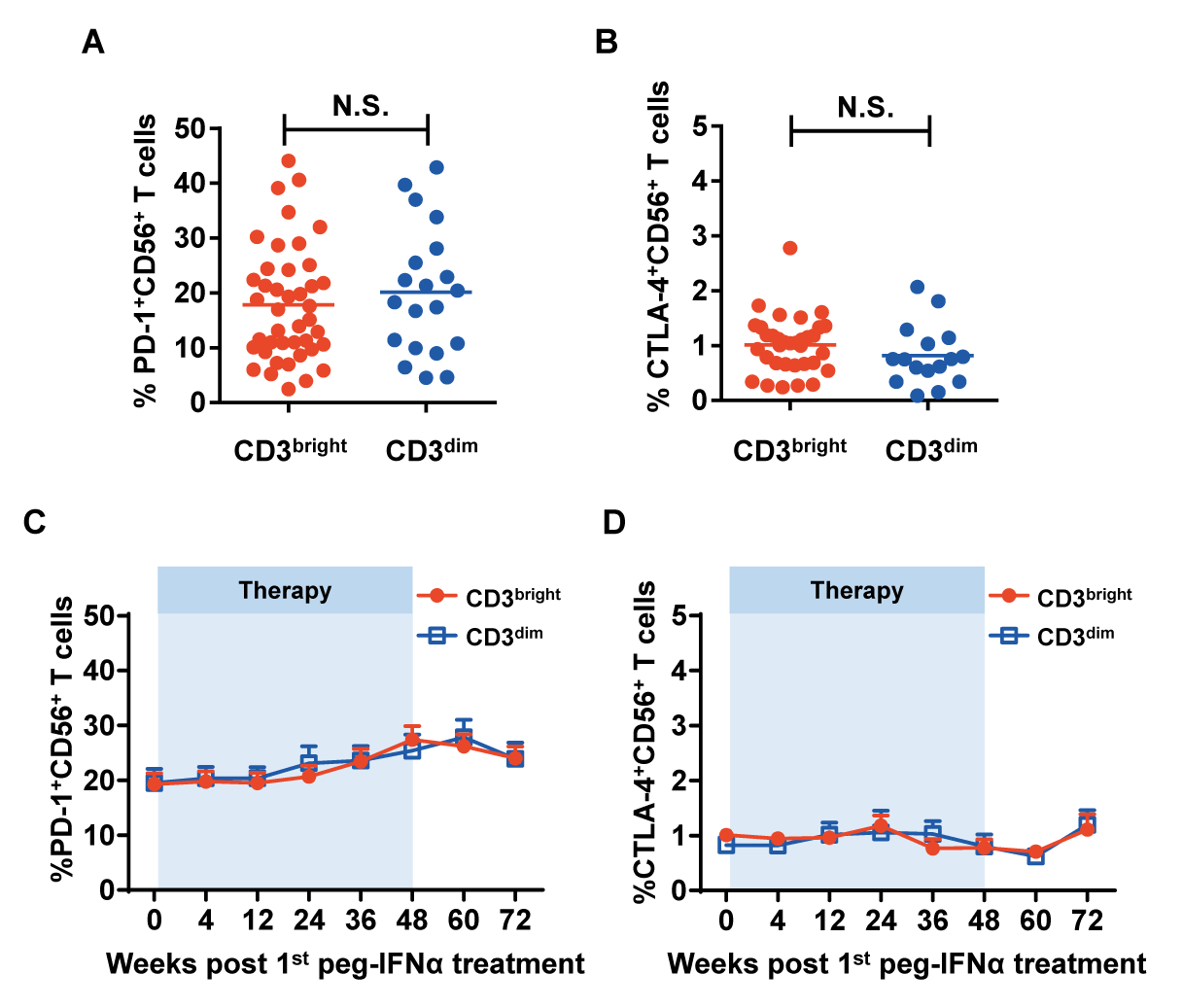


**Supplementary Fig. S4. CD3^bright^CD56^+^ T cells expressed similar levels of other inhibitory molecules compared to CD3^dim^CD56^+^ T cells.**


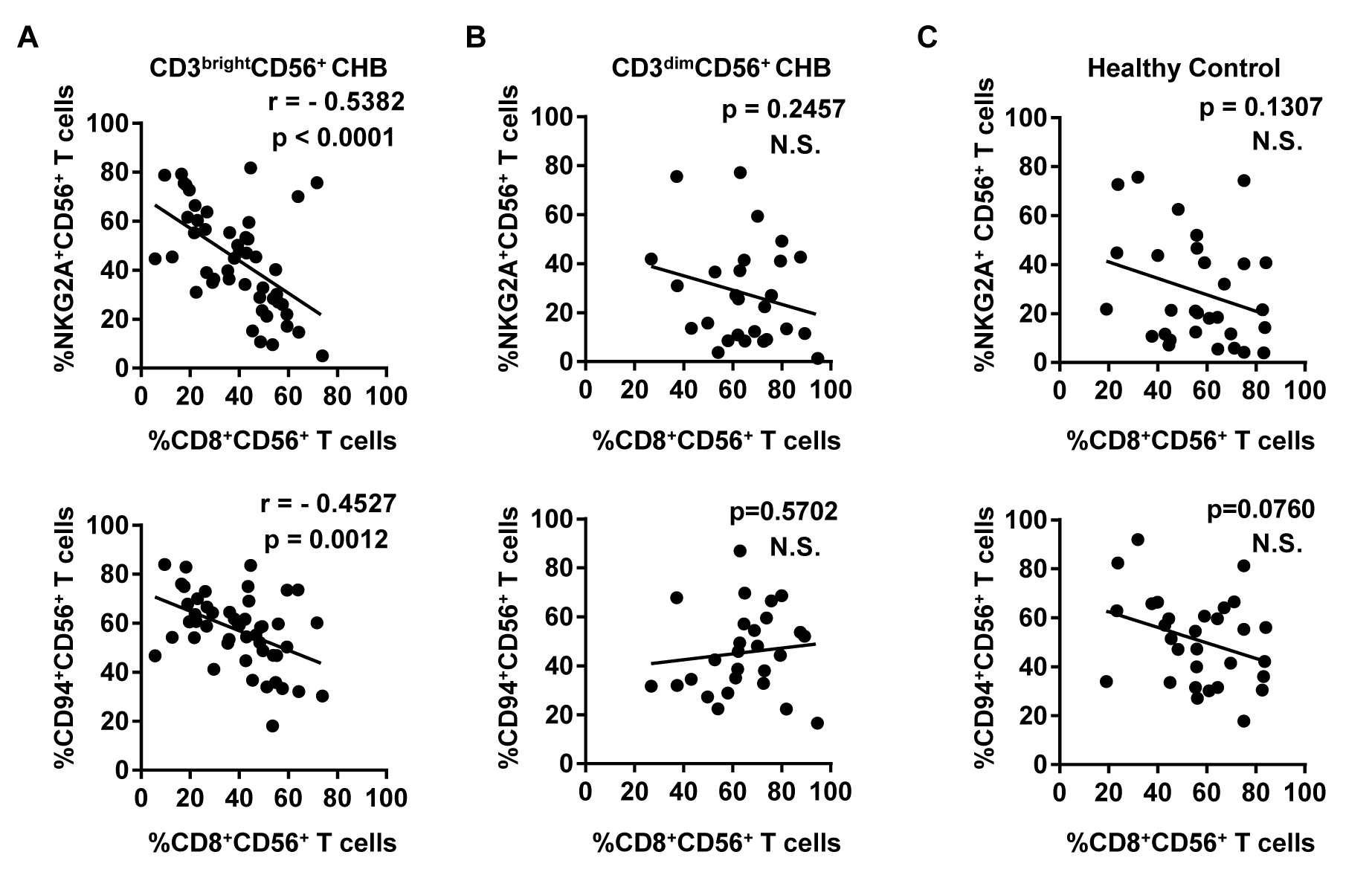


**Supplementary Fig. S5. The inverse correlation between NKG2A/CD94 and CD8 expression on CD3^bright^CD56^+^ T cells.**


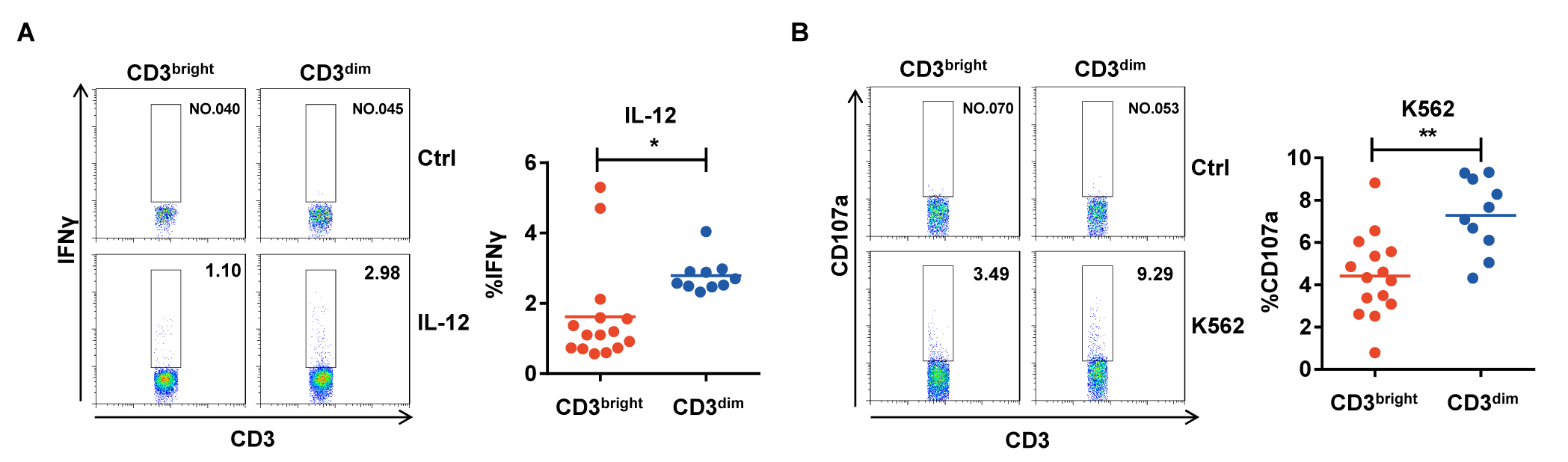


**Supplementary Fig. S6. CD3^bright^CD56^+^ T cells produce less** **IFNγ and CD107a compared to CD3^dim^CD56^+^ T cells.**


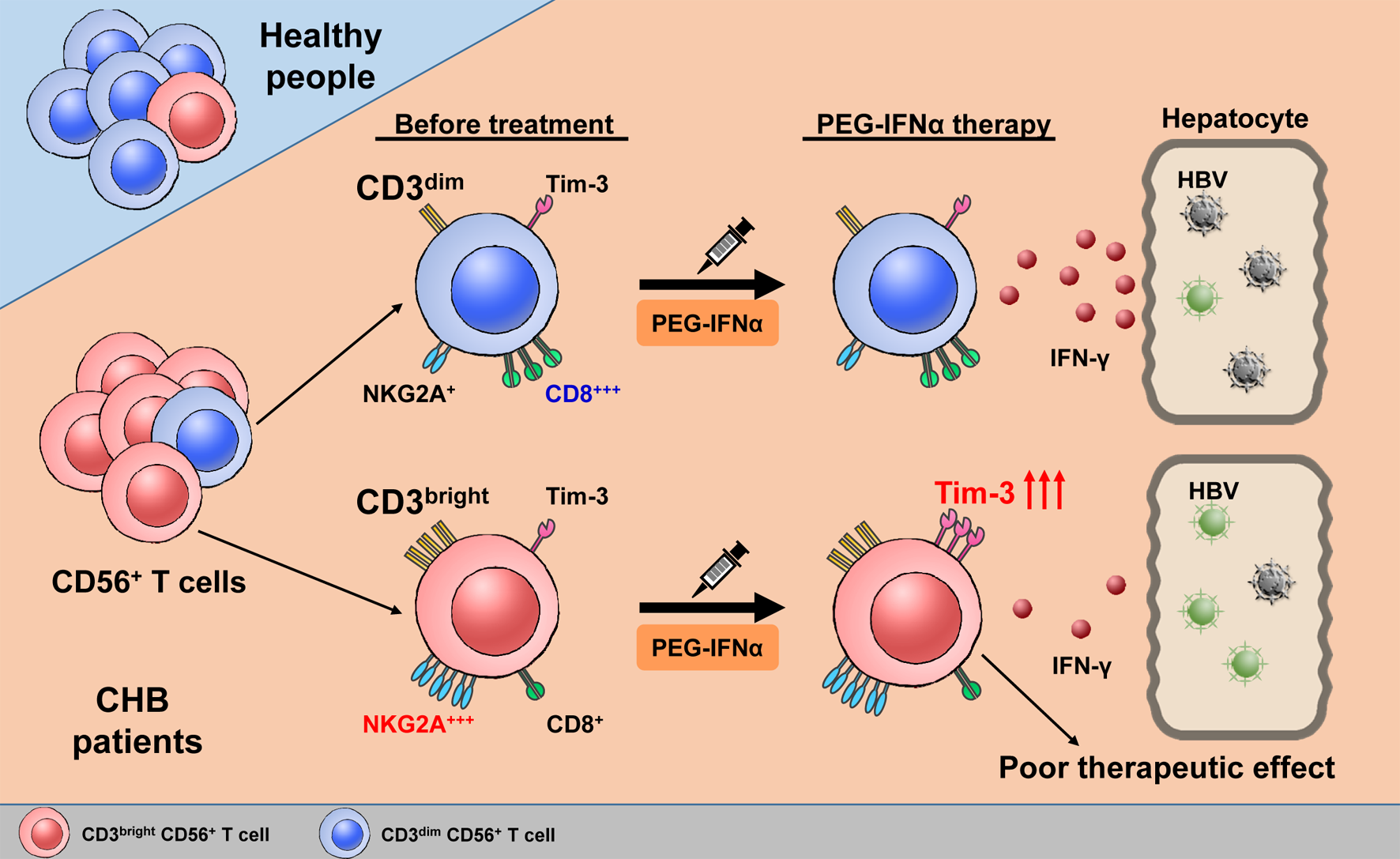


**Supplementary Fig. S7. CD3^bright^CD56^+^ T cells associate with pegylated interferon-alpha** **treatment nonresponse in HBV patients.**


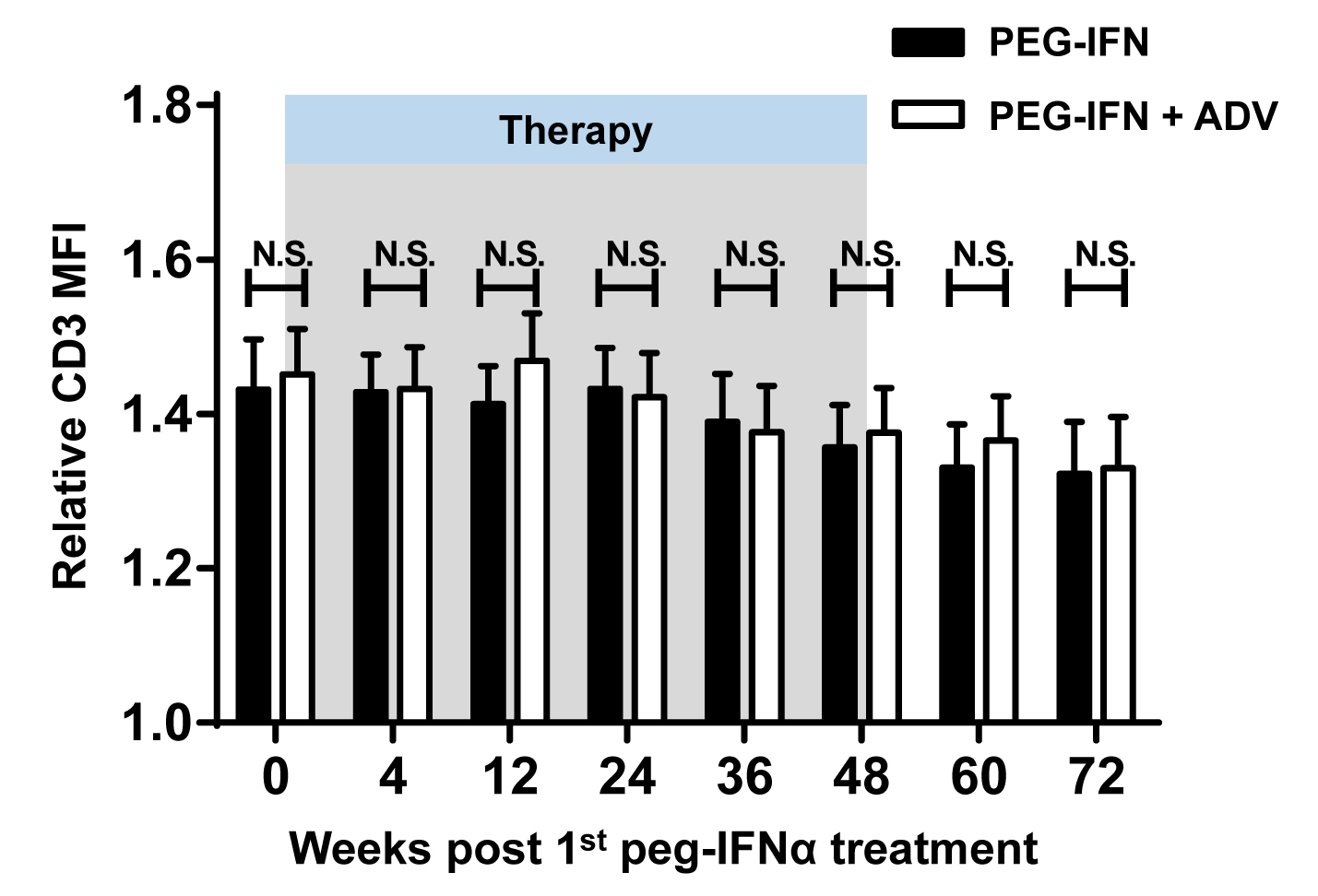


**Supplementary Fig. S8. The relative CD3 MFI of CD56^+^ T cells from patients treated with either PEG-IFNα or PEG-IFNα plus ADV at the indicated time points since peg-IFNα treatment.**


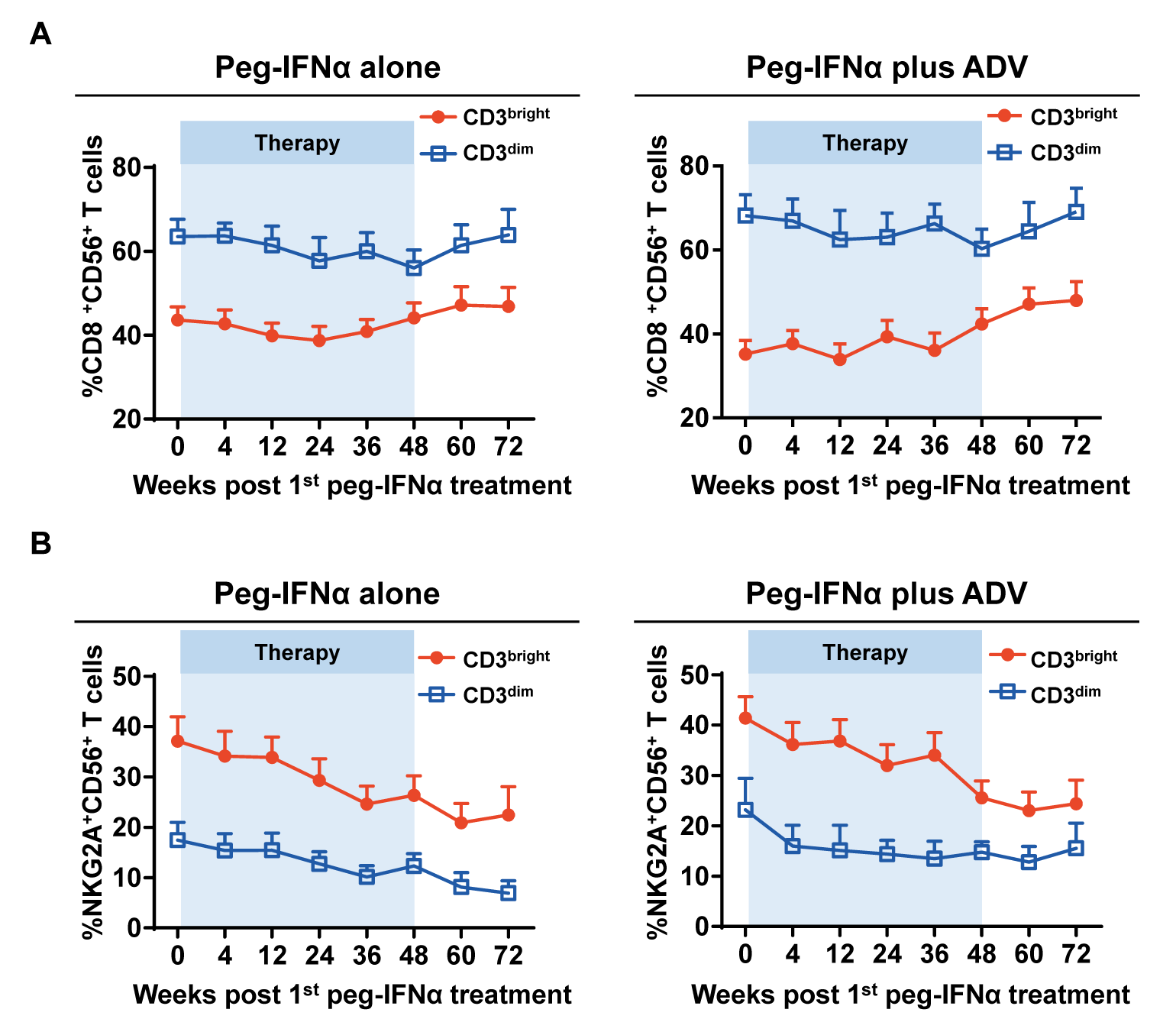


**Supplementary Fig. S9. CD8 and NKG2A expression levels on CD3^bright^ CD56^+^T and CD3^dim^ CD56^+^T cells from** **CHB patients since peg-IFNα treatment with or without ADV.**

**Supplementary Figure Legends**

**Supplementary Fig. S1. Gating Strategy.** (**A**) CD45 was first labeled to identify the lymphocyte population. Fluorescent antibodies against anti-CD3 and anti-CD56 were used to identify CD56^+^ T cells in CHB patient PBMCs. (**B**) PI was used to exclude dead lymphocytes.

**Supplementary Fig. S2. ROC curve of relative CD3 MFI.** ROC curve determined the cut-off of relative CD3 MFI (CD56^+^ T cell/ CD56^-^ T cells) from CHB patients based on healthy controls. When relative CD3 MFI was 1.33, the sensitivity and specificity of ROC curve obtained the optimal results. Thus, the criteria to separate individuals into CHB patients harboring CD3^bright^ CD56^+^ T cells or harboring CD3^dim^ CD56^+^ T cells is quantitative defined.

**Supplementary Fig. S3. CD3^bright^CD56^+^ T cells expressed similar levels of other functional molecules compared to CD3^dim^CD56^+^ T cells.** (**A-C**) CD3^bright^CD56^+^ T cells expressed similar levels of CD69 (A), CD57 (B), and CD16 (C) compared to CD3^dim^CD56^+^ T cells. (**D-F**) CD3^bright^CD56^+^ T cells (red) expressed similar levels of CD69 (D), CD57 (E), and CD16 (F) compared to CD3^dim^CD56^+^ T cells (blue) since peg-IFNα treatment. Error bars, s.e.m. N.S., not significant.

**Supplementary Fig. S4. CD3^bright^CD56^+^ T cells expressed similar levels of other inhibitory molecules compared to CD3^dim^CD56^+^ T cells.** (**A, B**) CD3^bright^CD56^+^ T cells expressed similar levels of PD-1 (A) and CTLA-4 (B) compared to CD3^dim^CD56^+^ T cells. (**C, D**) CD3^bright^CD56^+^ T cells (red) expressed similar levels of PD-1 (C) and CTLA-4 (D) compared to CD3^dim^CD56^+^ T cells (blue) since peg-IFNα treatment. Error bars, s.e.m. N.S., not significant.

**Supplementary Fig. S5. The inverse correlation between NKG2A/CD94 and CD8 expression on CD3^bright^CD56^+^ T cells.** (**A**) NKG2A/CD94 was inversely correlated with CD8 expression on CD3^bright^CD56^+^ T cells in CHB patients before treatment. (**B**) The relationship between NKG2A/CD94 and CD8 expression on CD3^dim^CD56^+^ T cells in CHB patients before treatment. (**C**) The relationship between NKG2A/CD94 and CD8 expression on CD56^+^ T cells in healthy controls. N.S., not significant.

**Supplementary Fig. S6. CD3^bright^CD56^+^ T cells produce less** **IFNγ and CD107a compared to CD3^dim^CD56^+^ T cells.** (**A**) Fresh PBMCs were stimulated with or without IL-12 for 16 h, and IFNγ production was determined as above. Statistical data is shown. (**B**) CD107a expression was determined in fresh PBMCs and K562 cells co-cultured at a 10:1 ratio for 4 h. Statistical data is shown. Error bars, s.e.m. **P* < 0.05, ***P* < 0.01.

**Supplementary Fig. S7. CD3^bright^CD56^+^ T cells associate with pegylated interferon-alpha** **treatment nonresponse in HBV patients. A majority of CHB patients preferentially harbored this CD3^bright^CD56^+^ T cell population. CD3^bright^CD56^+^ T cells had higher expression of inhibitory receptor NKG2A, lower CD8 expression and** lower IFNγ secretion, which is considered to be the main driving force of the antiviral response against HBV. Peg-IFNα treatment did not improve the CD3^bright^CD56^+^ T cell inhibitory state. **When CHB patients were treated with** peg-IFNα, Tim-3 expression on **CD3^bright^CD56^+^ T cells increased rapidly. As a result, t**he presence of **CD3^bright^CD56^+^ T cell**s in a CHB patient associated with poor therapeutic responses to peg-IFNα treatment.

**Supplementary Fig. S8. The relative CD3 MFI of CD56^+^ T cells from patients treated with either PEG-IFNα or PEG-IFNα plus ADV at the indicated time points since peg-IFNα treatment.** The relative CD3 MFI of CD56^+^ T cells from patients treated with either PEG-IFNα or PEG-IFNα plus ADV was determined at the indicated time points since peg-IFNα treatment.

**Supplementary Fig. S9. CD8 and NKG2A expression levels on CD3^bright^ CD56^+^T and CD3^dim^ CD56^+^T cells from** **CHB patients since peg-IFNα treatment with or without ADV. (A)** CD8 expression on CD3^bright^CD56^+^ (red) and CD3^dim^CD56^+^ (blue) T cells from their respective CHB patient groups at the indicated time points since peg-IFNα treatment alone (left) or peg-IFNα in combination with ADV (right). **(B)** NKG2A expression on CD3^bright^CD56^+^ (red) and CD3^dim^CD56^+^ (blue) T cells from their respective CHB patient groups at the indicated time points since peg-IFNα treatment alone (left) or peg-IFNα in combination with ADV (right). ADV, adefovir dipivoxil.
